# Highlights from the 11th ISCB Student Council Symposium 2015: Dublin, Ireland. 10 July 2015

**DOI:** 10.1186/s12859-016-0901-4

**Published:** 2016-02-25

**Authors:** Katie Wilkins, Mehedi Hassan, Margherita Francescatto, Jakob Jespersen, R. Gonzalo Parra, Bart Cuypers, Dan DeBlasio, Alexander Junge, Anupama Jigisha, Farzana Rahman, Griet Laenen, Sander Willems, Lieven Thorrez, Yves Moreau, Nagarajan Raju, Sonia Pankaj Chothani, C. Ramakrishnan, Masakazu Sekijima, M. Michael Gromiha, Paddy J Slator, Nigel J Burroughs, Przemysław Szałaj, Zhonghui Tang, Paul Michalski, Oskar Luo, Xingwang Li, Yijun Ruan, Dariusz Plewczynski, Giulia Fiscon, Emanuel Weitschek, Massimo Ciccozzi, Paola Bertolazzi, Giovanni Felici, Bart Cuypers, Pieter Meysman, Manu Vanaerschot, Maya Berg, Hideo Imamura, Jean-Claude Dujardin, Kris Laukens, Westa Domanova, James R. Krycer, Rima Chaudhuri, Pengyi Yang, Fatemeh Vafaee, Daniel J. Fazakerley, Sean J. Humphrey, David E. James, Zdenka Kuncic

**Affiliations:** Plant Pathology and Plant-Microbe Biology Section, School of Integrative Plant Science, Cornell University, Ithaca, NY USA; Graduate Field of Computational Biology, Cornell University, Ithaca, NY USA; School of Computing & Mathematics, University of South Wales, Cardiff, UK; Department of Genome Biology for Neurodegenerative Diseases, German Center for Neurodegenerative Diseases (DZNE) within the Helmholtz Association, ᅟ, Germany; Department of Surgery, Massachusetts General Hospital, The Broad Institute of MIT and Harvard, Boston, USA; Department of Systems Biology, Technical University of Denmark, Kemitorvet, Denmark; Protein Physiology Lab, Facultad de Ciencias Exactas y Naturales, Universidad de Buenos Aires, Buenos Aires, Argentina; Molecular Parasitology Unit (MPU), Institute of Tropical Medicine, Nationalestraat 155, 2000 Antwerp, Belgium; Advanced Database Research and Modeling (ADReM) research group, University of Antwerp, Middelheimlaan 1, 2020 Antwerpen, Belgium; Department of Computer Science, University of Arizona, Tucson, AZ 84721 USA; Department of Veterinary Clinical and Animal Sciences, Center for non-coding RNA in Technology and Health, University of Copenhagen, Copenhagen, Denmark; University College Dublin, Dublin, Ireland; Department of Electrical Engineering (ESAT), STADIUS Center for Dynamical Systems, Signal Processing and Data Analytics, KU Leuven, Leuven, Belgium; iMinds Medical IT Department, Leuven, Belgium; Scientific Institute of Public Health (WIV-ISP), Platform of Biotechnology and Molecular Biology (PBB), Brussels, Belgium; Department of Development and Regeneration @ Kulak, KU Leuven, Kortrijk, Belgium; Department of Biotechnology, Bhupat and Jyoti Metha School of Biosciences, Indian Institute of Technology Madras, Chennai, 600036 Tamilnadu India; Philips Research North America, 345 Scarborough Road, Briarcliff Manor, NY 10510 USA; Global Scientific Information and Computing Center (GSIC), Tokyo Institute of Technology, 2-12-1 Ookayama, Meguro-ku, Tokyo, 152-8550 Japan; Systems Biology Centre, University of Warwick, Senate House, Coventry, UK; Systems Biology Doctoral Training Centre, University of Warwick, Senate House, Coventry, UK; Center for Bioinformatics and Data Analysis, Medical University of Bialystok, ᅟ, Poland; I-BioStat, Hasselt University, ᅟ, Belgium; The Jackson Laboratory for Genomic Medicine, Farmington, USA; Department of Genetics and Genome Sciences, UConn Health, ᅟ, USA; Centre of New Technologies, Warsaw University, ᅟ, Poland; Department of Computer, Control and Management Engineering, Sapienza University, 00185 Rome, Italy; Institute for Systems Analysis and Computer Science, National Research Council, 00185 Rome, Italy; Department of Engineering, International University UNINETTUNO, 00186 Rome, Italy; Istituto Superiore di Sanità, 00161 Rome, Italy; Advanced Database Research and Modeling (ADReM), University of Antwerp, Antwerp, Belgium; Biomedical informatics research center Antwerp (biomina), Antwerp, Belgium; Molecular Parasitology Unit, Department of Biomedical Sciences, Institute of Tropical Medicine, Antwerp, Belgium; Department of Biomedical Sciences, University of Antwerp, Antwerp, Belgium; Department of Microbiology and Immunology, Columbia University Medical Center, New York, NY USA; Charles Perkins Centre, The University of Sydney, ᅟ, NSW 2006 Australia; School of Physics, The University of Sydney, Sydney, NSW 2006 Australia; School of Molecular Bioscience, The University of Sydney, ᅟ, NSW 2006 Australia; Sydney Medical School, The University of Sydney, ᅟ, NSW 2006 Australia; School of Mathematics and Statistics, The University of Sydney, ᅟ, NSW 2006 Australia; NIH/NIEHS, ᅟ, NC 27709 USA; Department of Proteomics and Signal Transduction, Max Planck Institute for Biochemistry, Martinsried, 82152 Germany

## Abstract

A1 Highlights from the eleventh ISCB Student Council Symposium 2015

Katie Wilkins, Mehedi Hassan, Margherita Francescatto, Jakob Jespersen, R. Gonzalo Parra, Bart Cuypers, Dan DeBlasio, Alexander Junge, Anupama Jigisha, Farzana Rahman

O1 Prioritizing a drug’s targets using both gene expression and structural similarity

Griet Laenen, Sander Willems, Lieven Thorrez, Yves Moreau

O2 Organism specific protein-RNA recognition: A computational analysis of protein-RNA complex structures from different organisms

Nagarajan Raju, Sonia Pankaj Chothani, C. Ramakrishnan, Masakazu Sekijima; M. Michael Gromiha

O3 Detection of Heterogeneity in Single Particle Tracking Trajectories

Paddy J Slator, Nigel J Burroughs

O4 3D-NOME: 3D NucleOme Multiscale Engine for data-driven modeling of three-dimensional genome architecture

Przemysław Szałaj, Zhonghui Tang, Paul Michalski, Oskar Luo, Xingwang Li, Yijun Ruan, Dariusz Plewczynski

O5 A novel feature selection method to extract multiple adjacent solutions for viral genomic sequences classification

Giulia Fiscon, Emanuel Weitschek, Massimo Ciccozzi, Paola Bertolazzi, Giovanni Felici

O6 A Systems Biology Compendium for Leishmania donovani

Bart Cuypers, Pieter Meysman, Manu Vanaerschot, Maya Berg, Hideo Imamura, Jean-Claude Dujardin, Kris Laukens

O7 Unravelling signal coordination from large scale phosphorylation kinetic data

Westa Domanova, James R. Krycer, Rima Chaudhuri, Pengyi Yang, Fatemeh Vafaee, Daniel J. Fazakerley, Sean J. Humphrey, David E. James, Zdenka Kuncic

## A1 Highlights from the eleventh ISCB Student Council Symposium 2015

### Katie Wilkins^1,2^, Mehedi Hassan^3^, Margherita Francescatto^4^, Jakob Jespersen^5,6^, R. Gonzalo Parra^7^, Bart Cuypers^8,9^, Dan DeBlasio^10^, Alexander Junge^11^, Anupama Jigisha^12^, Farzana Rahman^3^

#### ^1^Plant Pathology and Plant-Microbe Biology Section, School of Integrative Plant Science, Cornell University, Ithaca, NY, USA; ^2^Graduate Field of Computational Biology, Cornell University, Ithaca, NY, USA; ^3^School of Computing & Mathematics, University of South Wales, Cardiff, UK; ^4^Department of Genome Biology for Neurodegenerative Diseases, German Center for Neurodegenerative Diseases (DZNE) within the Helmholtz Association, Germany; ^5^Department of Surgery, Massachusetts General Hospital, The Broad Institute of MIT and Harvard, Boston, USA; ^6^Department of Systems Biology, Technical University of Denmark, Kemitorvet, Denmark; ^7^Protein Physiology Lab, Facultad de Ciencias Exactas y Naturales, Universidad de Buenos Aires, Buenos Aires, Argentina; ^8^Molecular Parasitology Unit (MPU), Institute of Tropical Medicine, Nationalestraat 155, 2000 Antwerp, Belgium; ^9^Advanced Database Research and Modeling (ADReM) research group, University of Antwerp, Middelheimlaan 1, 2020 Antwerpen, Belgium; ^10^University of Arizona, Department of Computer Science, Tucson, AZ 84721, USA; ^11^Center for non-coding RNA in Technology and Health, Department of Veterinary Clinical and Animal Sciences, University of Copenhagen, Copenhagen, Denmark; ^12^University College Dublin, Dublin, Ireland

##### **Correspondence:** Farzana Rahman (frahman@iscb.org) – School of Computing & Mathematics, University of South Wales, Cardiff, UK

Katie Wilkins and Farzana Rahman contributed equally to this work.

**Abstract**

This report summarizes the scientific content and activities of the annual symposium organized by the Student Council of the International Society for Computational Biology (ISCB), held in conjunction with the Intelligent Systems for Molecular Biology (ISMB) / European Conference on Computational Biology (ECCB) conference in Dublin, Ireland on July 10, 2015.

**About the Student Council and the symposium**

The Student Council (SC), part of the International Society for Computational Biology (ISCB), aims at nurturing and assisting the next generation of computational biologists. Our membership and leadership are composed of volunteer students and post-docs in computational biology and related fields. The main goal of our organization is to offer networking and soft skill development opportunities to our members.

The Student Council Symposium (SCS) takes place every year, directly preceding the ISMB/ECCB conferences. SCS 2015 marked the eleventh consecutive edition of the event [1-7].

**Meeting format**

The Student Council Symposium is a one-day event. Repeating a successful event from previous years, SCS 2015 opened with a scientific speed dating session. During this session our delegates find a partner to introduce themselves to and then learn about each other’s scientific backgrounds and interests. After ten minutes everyone switches partners, and this goes on until the allotted time runs out. The traditional scientific component of the meeting consisted of two keynote presentations by senior researchers, twelve student presentations, and a poster session.

At SCS 2015, Dr. Ruth Nussinov (National Cancer Institute, USA and Tel Aviv University, Israel) and Dr. Des Higgins (Conway Institute, University College Dublin, Ireland) generously agreed to deliver the keynote addresses. The symposium also included two short presentations on open science. The first, about publishing in the digital era, was given by Dr. Michael Markie, a faculty member at our institutional partner F1000 (UK). The second, about data sharing, was given by Dr. Robert Davey, a group leader with our institutional partner The Genome Analysis Center (UK).

Students submitted 100 abstracts to SCS 2015, which were peer-reviewed by 25 independent reviewers. Approximately 75 abstracts were accepted for poster presentations and 12 of these were also accepted for oral presentation. Extended abstracts of oral presentations are included in this report. All abstracts are available online in the SCS 2015 booklet (http://scs2015.iscbsc.org/scs2015-booklet).

**Keynotes**

The day opened with Dr. Ruth Nussinov’s keynote, in which she explained that Ras GTPase proteins involved in signal transduction include oncogenes such as KRas4B. While little is currently know about the mechanism by which the different mutations of KRas4B lead to cancer, Dr. Nussinov revealed that different mutations are differentially associated with different types of cancer. She believes that “structural biology, computations and experiment, are uniquely able to tackle” this question.

In the afternoon, Dr. Des Higgins began his keynote presentation with a history of multiple alignment algorithms. He then presented his newest alignment program, Clustal Omega, designed to align large numbers of sequences quickly and accurately.

**Student presentations**

The student presentations were begun by Griet Laenen, who shared a new method for identifying drug targets [8]. Each putative target is ranked based on the transcriptional response of functionally related genes and based on the structural similarity of the drug to proteins know to interact with the putative target. On a ChEMBL-derived test set, AUC values of up to 90 % were achieved.

Nagarajan Raju reported the identification of different modes of protein-RNA interaction in different organisms, based on structural analysis and molecular dynamics simulation [9].

The long reads available from third generation sequencing methods are a valuable resource, but can complicate mapping because of their higher error rate. Hybrid methods use more accurate short reads to correct these errors but current methods for doing so are either too slow or less accurate on larger genomes. Giles Miclotte presented the Jabba hybrid error correction method which uses corrected de Bruijin graphs to achieve comparable performance on small genomes while still performing well for larger genomes [10].

Drawing robust biological conclusions from stochastic single particle tracking trajectories is particularly difficult when particle movement is heterogeneous, such as in the plasma membrane. Paddy Slator described a method for dealing with heterogeneity by analyzing multiple models of heterogeneity and calculating model selection statistics to identify the most likely of these models, accounting for noisy data. This method was tested on several real data sets [11].

Although we often think of DNA as a two-dimensional sequence, the development of the high-throughput ChIA-PET method for identifying all physical contacts between distant loci is enabling modeling of DNA as a three-dimensional structure. Przemysław Szałaj presented 3D-NOME, a new computational method for modelling the three-dimensional structure of the genome [12]. Based on both ChIA-PET data and known interactions of CTCF and RNAPII, 3D-NOME builds an initial model of the nucleosome in a bottom-up fashion and then uses Monte Carlo simulations to reconstruct each level of structure in a top-down fashion.

Epigenome-wide association studies allow the high-throughput identification of epigenetic markers that contribute to human disease. Charles Edmund Breeze presented eFORGE, a tool for identifying the cell type specificity of differentially methylated positions significantly associated with disease. eFORGE identifies cell types of interest based on enrichment of overlap between significant differentially methylated positions and DNase I hypersensitive sites in each cell type, compared to matched, randomly generated background sets.

Jonas Ibn-Salem used Hi-C chromatin conformation to show that paralogous genes share more enhancer elements with one another and is more likely to occur in the same topological association domain then matched, randomly selected gene pairs. This suggests that paralogs share common regulation because and cluster within the three-dimensional chromatin architecture.

As the number of genome sequences available increases, it becomes more challenging for biologists to characterize the data and use it to classify sequenced organisms. Giulia Fiscon presented a new feature extraction method for solving this problem, which she integrated into a classifier used to assign virus specimens to viral species [13].

RIG-I-like receptors (RLRs) are an important component of the immune response to viruses, detecting viral RNA and triggering the production of type I interferons. Robin van der Lee identified 10 features common to RLR pathway components and used these features to predict a role in the RLR pathway for 187 novel genes. RNAi knock-down screens showed that almost half of these genes had an impact on production of type I interferon, IFN*β* [14].

Only four drugs for treating the lethal disease visceral leishmaniasis are available and the resistance is emerging rapidly in the causal parasite, *Leishmania donovani*. Bart Cuypers shared a new database, integrating the genomic, epigenomic, transcriptomic, proteomic, metabolomics, and phenotype data available for *L. donovani*. This database is coupled with many novel data-analysis tools, such as one used to find patterns in different omics data across different conditions [15].

In the final oral presentation of the day, Westa Domanova showed that temporal data could be used to distinguish the activity of different kinases, even those sharing the same consensus motif [16].

**Award winners**

Thanks to the generous contribution of the Swiss Institute of Bioinformatics, two travel fellowships were awarded to Alexander Monzon and Marek Cmero. Thanks to the generous contributions of BioMed Central, an additional two travel fellowships were awarded to Westa Domanova and Güngör Budak. Finally, the generous contributions of F1000 enabled the award of a fifth travel fellowship to Giulia Fiscon. The ISCB Student Council also awarded an *Inspiring Youth* travel fellowship to high school student Prathik Naidu.

Based on the votes of the SCS delegates, a judging committee awarded three speakers with one best oral and two best poster presentation awards. The Best Presentation Award went to Griet Laenen for her work “Prioritizing a drug’s targets using both gene expression and structural similarity”. The first place poster award went to Robin van der Lee for his poster, “Systematic integration of molecular signatures identifies novel components of the antiviral RIG-I-like receptor pathway”. The second place poster award went to Jonas Ibn-Salem for his poster “Co-regulation of human paralog genes in the three-dimensional chromatin architecture”. These awards were enabled by the generous support of Oxford University Press.

F1000 awards were also made possible by the generosity of that journal and these were awarded to Bart Cuypers, Giles Miclotte, and Charles Breeze for having posters and presentations that were rated highly by SCS delegates.

**Student Council activities at ISMB**

In addition to the one day Student Council symposium, the Student Council organizes several activities available to all students and young researchers participating in ISMB/ECCB.

**Interactive job postings and student council lounge**

To facilitate the interaction between job seekers and job advertisers, the Student Council organizes an interactive job postings board during ISMB/ECCB. Job offers can be attached to the posting board available at the SC booth in the exhibitors’ hall. The SC booth also served as a lounge area where students and young researchers from ISMB/ECCB could network with one another and meet members and leaders of the Student Council.

**Career Central workshop**

The goal of Student Council Career Central is to expose students to the experiences and success stories of senior researchers. To this end, the Student Council collaborated with the Junior PI group and COBE COSI to organize an Applied Knowledge Exchange Sessions workshop dedicated to career development. The Student Council invited the first speaker of the session, Dr. Mick Watson, Director of ARK-Genomics, The Roslin Institute, University of Edinburgh, UK. He focused his advice on how to give an effective elevator pitch and gave attendees to practice his advice by giving their elevator pitches to one another.

**Conclusions**

This year’s number of submissions and participants increased by about a third and a half respectively over last year’s symposium. It seems likely that this is due in part to greater availability of funding and easier acquisition of visas in Europe for many participants. The increased number of participants, the participants’ enthusiastic response to the networking opportunities and talks on open access science, and the high quality keynotes, student presentations, and posters, made this year’s Student Council Symposium a great success.

**Acknowledgements**

Because of space constraints we are unable to mention in this publication all the volunteers whose contributions make the Student Council Symposium a reality every year. Our recognition and appreciation goes out to all of them, since without their support the organization of such an event would simply not be possible.

We would like to thank ISCB Executive Director Diane Kovats, ISCB Conferences Director Steven Leard, ISMB Conference Administrator Pat Rodenburg, ISMB programmer Jeremy Henning, and ISCB Administrative Support Suzi Smith for their logistical support and invaluable advice. Furthermore, we thank the ISCB Board of Directors for their continued support of the ISCB Student Council in general and the Student Council Symposium in particular.

We are greatly indebted to ISMB 2015 conference chairs Dr. Alex Bateman and Dr. Janet Kelso for giving us the opportunity to organize the Student Council Symposium 2015 in Dublin.

The Student Council would also like to thank our keynote speakers Dr. Ruth Nussinov and Dr. Des Higgins who generously donated their valuable time by delivering keynote addresses.

The Symposium would not be possible without the financial support of our generous sponsors. We would like to thank BioMed Central, Oxford University Press, Swiss Institute of Bioinformatics, F1000, Bina, and The Genome Analysis Center for their contributions.

We are very grateful to all the volunteer reviewers for their work on ensuring the quality of the scientific program, and to the program and travel fellowship committees for coordinating the reviewing effort.

Finally, we thank all Student Council members that have spent countless hours organizing all aspects of SCS 2015 to ensure its success.

**References**

1. Rahman F, Wilkins K, Jacobsen A, Junge A, Vicedo E, DeBlasio D, Jigisha A, Di Domenico T: Highlights from the tenth ISCB Student Council Symposium 2014. BMC bioinformatics 2015, 16(Suppl 2):A1.

2. Di Domenico T, Prudence C, Vicedo E, Guney E, Jigisha A, Shanmugam A: Highlights from the ISCB Student Council Symposium 2013. BMC bioinformatics 2014, 15(Suppl 3):A1-A1.

3. Abeel T, de Ridder J, Peixoto L: Highlights from the 5(th) International Society for Computational Biology Student Council Symposium at the 17(th) Annual International Conference on Intelligent Systems for Molecular Biology and the 8(th) European Conference on Computational Biology. BMC bioinformatics 2009, 10(13).

4. Klijn C, Michaut M, Abeel T: Highlights from the 6th International Society for Computational Biology Student Council Symposium at the 18th Annual International Conference on Intelligent Systems for Molecular Biology. BMC bioinformatics 2010, 11(Suppl 10):4.

5. Goncearenco A, Grynberg P, Botvinnik O, Macintyre G, Abeel T: Highlights from the Eighth International Society for Computational Biology (ISCB) Student Council Symposium 2012. BMC bioinformatics 2012, 13(Suppl 18):A1.

6. Peixoto L, Gehlenborg N, Janga S: Highlights from the Fourth International Society for Computational Biology Student Council Symposium at the Sixteenth Annual International Conference on Intelligent Systems for Molecular Biology. BMC bioinformatics 2008, 9(10).

7. Grynberg P, Abeel T, Lopes P, Macintyre G, Rubino L: Highlights from the Student Council Symposium 2011 at the International Conference on Intelligent Systems for Molecular Biology and European Conference on Computational Biology. BMC bioinformatics 2011, 12(11).

8. Laenen G, Willems S, Thorrez L , Moreau Y. Prioritizing a drug’s targets using both gene expression and structural similarity. BMC Bioinformatics 2015.

9. Raju N, Chothani SP, Ramakrishnan C, Sekijima M, Gromiha MM. Organism specific protein-RNA recognition: A computational analysis of protein-RNA complex structures from different organisms. BMC Bioinformatics 2015.

10. Slator PJ, Burroughs NJ. Detection of Heterogeneity in Single Particle Tracking Trajectories. BMC Bioinformatics 2015.

11. Szałaj P, Tang Z, Michalski P, Luo O, Li X, Ruan Y, Plewczynski. 3D-NOME: 3D NucleOme Multiscale Engine for data-driven modeling of three-dimensional genome architecture. BMC Bioinformatics 2015.

12. Fiscon G, Weitschek E, Ciccozzi M, Bertolazzi P, Felici G. A novel feature selection method to extract multiple adjacent solutions for viral genomic sequences classification. BMC Bioinformatics 2015.

13. van der Lee R, Feng Q, Langereis MA, ter Horst R, Szklarczyk R, Netea MG, Andeweg AC, van Kuppeveld FJM, Huynen MA. Integrative Genomics-Based Discovery of Novel Regulators of the Innate Antiviral Response. PLoS Comput Biol 2015, 11(10):e1004553, doi:10.1371/journal.pcbi.1004553.

14. Cuypers B, Mayesman P, Vanaerschot M, Berg M, Imamura H, Dujardin J-C, Laukens K. A Systems Biology Compendium for Leishmania donovani. BMC Bioinformatics 2015.

15. Domanova W, Krycer JR, Chaudhuri R, Yang P, Vafaee F, Fazakerley DJ, Humphrey SJ, James DE, Kuncic Z. Unravelling signal coordination from large scale phosphorylation kinetic data. BMC Bioinformatics 2015.

## O1 Prioritizing a drug’s targets using both gene expression and structural similarity

### Griet Laenen^1,2^, Sander Willems^3^, Lieven Thorrez^4^, Yves Moreau^1,2^

#### ^1^KU Leuven, Dept. of Electrical Engineering (ESAT), STADIUS Center for Dynamical Systems, Signal Processing and Data Analytics, Leuven, Belgium; ^2^iMinds Medical IT Dept., Leuven, Belgium; ^3^Scientific Institute of Public Health (WIV-ISP), Platform of Biotechnology and Molecular Biology (PBB), Brussels, Belgium; ^4^KU Leuven, Dept. of Development and Regeneration @ Kulak, Kortrijk, Belgium

##### **Correspondence:** Griet Laenen (griet.laenen@esat.kuleuven.be) – iMinds Medical IT Department, Leuven, Belgium

**Background**

The pharmaceutical industry is facing unprecedented pressure to increase its productivity. Attrition rates in the later stages of development have risen sharply, with toxicity and lack of efficacy being the main bottlenecks [1]. To address both these safety- and efficacy-related issues, a better understanding of the complex biological response to drug treatment is vital. Although many drugs exert their therapeutic activities through the modulation of multiple targets [2], these targets are often unknown and identification among the thousands of gene products remains difficult. We propose a computational method to support the identification of putative targets of a drug by means of a dual approach combining network diffusion of gene expression with chemical structure similarity.

**Methods**

The first component of our method prioritizes proteins as potential targets by integrating experimental gene expression data with prior knowledge on protein interactions [3, 4]. More specifically, genes are ranked based on the transcriptional response of functionally related genes by diffusing differential expression signals following treatment over a protein interaction network. In addition, drug-protein interactions can also be predicted from structural information. Building on the similar property principle, the second component of our method prioritizes proteins as drug targets based on the interaction with compounds structurally similar to the drug of interest. To this end compound-compound similarity scores are combined with compound-protein interaction scores. Both this structure-based and expression-based approach produce a genome-wide ranking of potential targets that can eventually be fused to obtain a single ranking.

**Results/Conclusion**

Our method has been evaluated on a test set of small molecule drugs for which the known targets were derived from ChEMBL [5]. AUC values of up to 90 % were obtained. These results indicate the predictive power of combining gene expression data and structural information for a drug of interest with known protein-protein and protein-compound interaction information respectively, to identify the targets of that drug. As such this dual method can aid in gaining a better knowledge of a candidate drug’s mode of action and its off-target effects and thus be of value in the drug development process.

**References**

1. Arrowsmith J, Harrison R. Drug repositioning: the business case and current strategies to repurpose shelved candidates and marketed drugs. In: Barratt MJ, Frail DE, editors. Drug Repositioning: Bringing New Life to Shelved Assets and Existing Drugs. Hoboken, NJ: John Wiley & Sons, Inc; 2012. p. 9-31.

2. Hopkins AL. Network pharmacology: the next paradigm in drug discovery. Nat. Chem. Biol. 2008; 4(11): 682-690.

3. Laenen G, Thorrez L, Börnigen D, Moreau Y. Finding the targets of a drug by integration of gene expression data with a protein interaction network. Mol. BioSyst. 2013; 9: 1676-1685.

4. Laenen G, Ardeshirdavani A, Moreau Y, Thorrez L. Galahad: a web server for drug effect analysis from gene expression. Nucl. Acids Res. 2015; 43: W208-212.

5. Bento AP, Gaulton A, Hersey A, Bellis LJ, Chambers J, Davies M, Krüger FA, Light Y, Mak L, McGlinchey S, Nowotka M, Papadatos G, Santos R, Overington JP. The ChEMBL bioactivity database: an update. Nucl. Acids Res. 2014; 42: D1083-D1090.

## O2 Organism specific protein-RNA recognition: A computational analysis of protein-RNA complex structures from different organisms

### Nagarajan Raju^1^, Sonia Pankaj Chothani^1,2^, C. Ramakrishnan^1^, Masakazu Sekijima^3^, M. Michael Gromiha^1^

#### ^1^Department of Biotechnology, Bhupat and Jyoti Metha School of Biosciences, Indian Institute of Technology Madras, Chennai 600036, Tamilnadu, India; ^2^Philips Research North America, 345 Scarborough Road, Briarcliff Manor, NY10510, USA; ^3^Global Scientific Information and Computing Center (GSIC), Tokyo Institute of Technology 2-12-1 Ookayama, Meguro-ku, Tokyo 152-8550, Japan

##### **Correspondence:** M. Michael Gromiha (gromiha@iitm.ac.in) – Department of Biotechnology, Bhupat and Jyoti Metha School of Biosciences, Indian Institute of Technology Madras, Chennai 600036, Tamilnadu, India

**Motivation**

Protein-RNA interactions play essential roles in many cellular processes. It is unclear whether same RNA binding proteins from different organisms show unique patterns or variations to recognize RNA. To address this issue, we have constructed 18 sets of same protein-RNA complexes belonging to different organisms and analyzed the interactions and interacting patterns using various sequence and structure based features [1].

**Results**

We have investigated the recognizing elements by grouping the protein chains into five organisms such as *E. coli*, *H. sapiens*, *S. cerevisiae*, archaea and thermophiles [2]. We observed that positively charged residues are highly preferred in *E. coli* whereas aromatic residues and polar residues show preference in *S. cerevisiae* and thermophiles, respectively. In case of RNA, adenine and uracil are highly preferred in *H. sapiens* and *S. cerevisiae,* respectively. The neighboring residues around the binding sites are unique in different organisms (Eg. Cys-His in *H. sapiens*, Ala-leu in *E. coli*, Ser-Arg in *S. cerevisiae*, Gly-Arg in archaea and Asn-Lys in thermophiles). Further, molecular dynamics simulations of aspartyl tRNA synthetase complexes from *E. coli*, *T. thermophilus* and *S. cerevisiae* revealed the similarities and differences in structurally equivalent binding site residues to understand the recognition mechanism.

**Conclusion**

Sequence and structural analysis along with MD simulation showed the variations in the interactions between protein and RNA to understand the recognition mechanism of protein-RNA complexes.

**References**

1. Raju N, Gromiha MM. Prediction of RNA binding residues: An extensive analysis based on structure and function to select the best predictor. PLoS One 2014; 9 (3): e91140.

2. Raju N, Chothani SP, Ramakrishnan C, Sekijima M, Gromiha MM. Structure based approach for understanding organism specific recognition of protein-RNA complexes. Biology Direct. 2015; 10: 8.

## O3 Detection of Heterogeneity in Single Particle Tracking Trajectories

### Paddy J Slator^1,2^, Nigel J Burroughs^1^

#### ^1^Systems Biology Centre, University of Warwick, Senate House, Coventry, UK; ^2^Systems Biology Doctoral Training Centre, University of Warwick, Senate House, Coventry, UK

##### **Correspondence:** Paddy J Slator (p.j.slator@warwick.ac.uk) – Systems Biology Doctoral Training Centre, University of Warwick, Senate House, Coventry, UK

**Background**

Single particle tracking trajectories are fundamentally stochastic, which makes the extraction of robust biological conclusions difficult. This is especially the case when trying to detect heterogeneous movement of molecules in the plasma membrane. This heterogeneity could be due to a number of biophysical processes such as: receptor clustering [1], traversing lipid microdomains [2, 3] or cytoskeletal barriers [4].

**Results**

**Fig. 1 (abstract O3).**
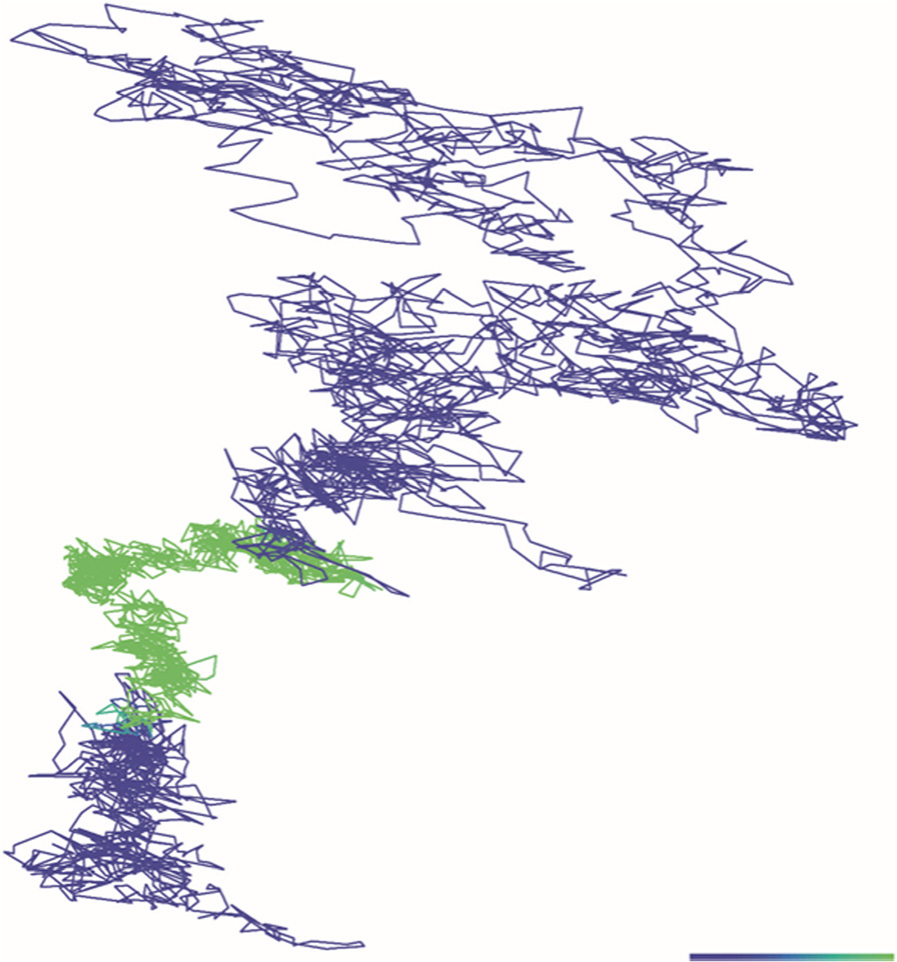
MCMC fit of two-state diffusion model with measurement noise to an LFA- 1 trajectory, from [5]. Colour denotes inferred diffusion state, with green slow diffusion and blue fast diffusion. Colorbar length 100 nm

Working in a Bayesian framework, we developed multiple models for heterogeneity, such as confinement in a harmonic potential well, switching between diffusion coefficients and diffusion in a fenced environment (or ‘hop’ diffusion). We implement these models using a Markov chain Monte Carlo (MCMC) methodology, developing algorithms that infer model parameters and hidden states from single trajectories. We also calculate model selection statistics, to determine the most likely model given the trajectory. Our methodology also accounts for measurement noise. For LFA-1 receptors diffusing on T cells we previously showed that 12-26 % of trajectories display clear switching between diffusive states, depending on treatment (example trajectory in Fig. 1) [5].

**Fig. 2 (abstract O3).**
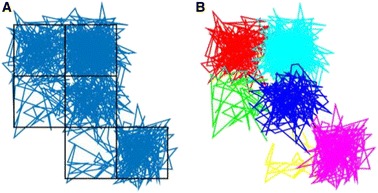
Detection of hopping diffusion in simulated data. **a** Simulated hop diffusion trajectory. **b** Hop diffusion model MCMC fit, with colours representing inferred trapping zone

Analysis of the motion of GM1 lipids bound to the cholera toxin B subunit in model membranes confirmed transient trapping in harmonic potential wells. We developed an algorithm which detects hopping diffusion, and validated on simulated data (Fig. 2).

We have also demonstrated that allowing for measurement noise is essential, as otherwise false detection of heterogeneity may be observed.

**Conclusions**

We have used Bayesian methodology to analyze single particle tracking trajectories. Rather than methods which rely on generic properties of Brownian motions, our approach allows us to test which biophysical model best fits a trajectory. With the continuing improvement in spatial and temporal resolution of trajectories, these methods will be important for biological interpretation of single particle tracking experiments.

**References**

1. Bray, D., Levin, M.D., Morton-Firth, C.J.: Receptor clustering as a cellular mechanism to control sensitivity. Nature 393(6680), 8588(1998).

2. Sahl, S.J., Leutenegger, M., Hilbert, M., Hell, S.W., Eggeling, C.: Fast molecular tracking maps nanoscale dynamics of plasma membrane lipids. Proceedings of the National Academy of Sciences 107(15), 6829-6834 (2010).

3. Lingwood, D., Simons, K.: Lipid rafts as a membrane-organizing principle. Science 327(5961), 46-50 (2010).

4. Kusumi, A., Suzuki, K.G.N., Kasai, R.S., Ritchie, K., Fujiwara, T.K.: Hierarchical mesoscale domain organization of the plasma membrane. Trends in Biochemical Sciences 36(11), 604-615 (2011).

5. Slator, P.J., Cairo, C.W., Burroughs, N.J.: Detection of Diffusion Heterogeneity in Single Particle Tracking Trajectories Using a Hidden Markov Model with Measurement Noise Propagation. PLOS ONE 10(10), 0140759 (2015).

## O4 3D-NOME: 3D NucleOme Multiscale Engine for data-driven modeling of three-dimensional genome architecture

### Przemysław Szałaj^1,2^, Zhonghui Tang^3^, Paul Michalski^3^, Oskar Luo^3^, Xingwang Li^3^, Yijun Ruan^3,4^, Dariusz Plewczynski^5^

#### ^1^Center for Bioinformatics and Data Analysis, Medical University of Bialystok, Poland; ^2^I-BioStat, Hasselt University, Belgium; ^3^The Jackson Laboratory for Genomic Medicine, Farmington, USA; ^4^Department of Genetics and Genome Sciences, UConn Health, USA; ^5^Centre of New Technologies, Warsaw University, Poland

##### **Correspondence:** Przemysław Szałaj (przemek.szalaj@uhasselt.be) – I-BioStat, Hasselt University, Belgium

**Background**

Human genome is folded into three-dimensional structures. The 3D organization of the genome is thought to facilitate compartmentalization, chromatin organization and spatial interaction of genes and their regulatory elements. Recently developed high-throughput ChIA-PET method allows us to capture the genome-wide map of physical contacts between distal genomic loci.

**Methods**

We present the 3D-NOME, a multi-scale computational engine we developed to model the 3D organization of the genome. First, we use a bottom-up approach to create a hierarchical model representing the nucleus based on the underlying data features. Then we use Monte Carlo simulations to sequentially reconstruct all the levels in a top-down manner, i.e. we reconstruct more general levels first and we use them to guide the simulation on the following levels. This approach allows us to efficiently model the chromatin folding on a level of whole chromosomes as well as single topological domains.

In our modeling we consider CTCF (which is long known to be responsible for chromatin weaving) and RNAPII (which activates genes transcription) interactions. Taken together these two protein factors provide a comprehensive map of human genome interactions. We also consider CTCF-motif orientations in order to obtain more reliable structures. The specificity of the ChIA-PET data allows us to model the shape of individual chromatin loops and their mutual interactions within topological domains boundaries.

We demonstrate the effectiveness of 3D-NOME in building 3D genome models at the kilobase resolution using ChIA-PET data of human B-lymphocytes (GM12878 cell line) [1].

**Conclusion**

In this work we describe both the model construction and simulation steps of our algorithm. We do also highlight main advantages of our approach compared to existing methods for genome architecture modeling. We hope that further refinement of 3D-NOME and application to additional ChIA-PET and other types of 3D genome mapping data will help to advance our understanding of genome structural organization and functioning.

**References**

1. Guoliang Li, Fullwood M J, Xu H, et al. (2010) ChIA-PET tool for comprehensive chromatin interaction analysis with paired-end tag sequencing. Genome Biol 11:R22.

## O5 A novel feature selection method to extract multiple adjacent solutions for viral genomic sequences classification

### Giulia Fiscon^1,2^, Emanuel Weitschek^2,3^, Massimo Ciccozzi^4^, Paola Bertolazzi^2^, Giovanni Felici^2^

#### ^1^Department of Computer, Control and Management Engineering, Sapienza University, 00185-Rome, Italy; ^2^Institute for Systems Analysis and Computer Science, National Research Council, 00185-Rome, Italy; ^3^Department of Engineering, International University UNINETTUNO, 00186-Rome, Italy; ^4^Istituto Superiore di Sanità, 00161-Rome, Italy

##### **Correspondence:** Giulia Fiscon (fiscon@dis.uniroma1.it) – Institute for Systems Analysis and Computer Science, National Research Council, 00185-Rome, Italy

**Background**

Leveraging improvements of next generation technologies, genome sequencing of several samples in different conditions led to an exponential growth of biological sequences. However, these collections are not easily treatable by biologists to obtain a thorough data characterization and require a high cost-time investment. Therefore, computing strategies and specifically automatic knowledge extraction methods that optimize the analysis focusing on what data are meaningful and should be sequenced are essential [1].

**Methods**

Here, we present a new feature-selection algorithm based on mixed integer programming methods [2] able to extract multiple and adjacent solutions for supervised learning problems applied to biological data. We focus on those problems where the relative position of a feature (i.e., nucleotide locus) is relevant. In particular, we aim to find sets of distinctive features, which are as close as possible to each other and which appear with the same required characteristics. Our algorithm adopts a fast and effective method to evaluate the quality of the extracted sets of features and it has been successfully integrated in a rule-based classification framework [3].

**Results**

Our algorithm has been applied to three viral datasets (i.e., Rhino-, Influenza-, Polyomaviruses [4-6]) and enables to extract all the alternative solutions of virus specimen to species assignments, by identifying portions of sequence that are discriminant, compact, and as shorter as possible.

To conclude, we succeeded in extracting a wide set of equivalent classification rules, focusing on short regions of sequences with high reliability and low computational time, in order to provide the biologists with short and highly informative genome parts to be sequenced, as well as a powerful instrument both scientifically and diagnostically, e.g., for automatic virus detection.

**References**

1. Chen JY and S. Lonardi S, Biological Data Mining, Chapman & Hall, 2010.

2. Bertolazzi P, Felici G, Festa P, Fiscon G, and Weitschek E. Integer programming models for feature selection: new extensions and a randomized solution algorithm. European Journal of Operational Research, 1-20, 2015, (accepted).

3. Weitschek E, Velzen R, Felici G, and Bertolazzi P. Blog 2.0: a software system for character-based species classification with dna barcode sequences. what it does, how to use it. Molecular ecology resources, 13(6):1043-1046, 2013.

4. Tapparel C, Junier T, Gerlach D, Cordey S, Van Belle S, Perrin L, Zdobnov EM, and Kaiser L. New complete genome sequences of human rhinoviruses shed light on their phylogeny and genomic features. BMC genomics, 8(1):224, 2007.

5. Weitschek W, Lo Presti A, Drovandi G, Felici G, Ciccozzi M, Ciotti M, and Bertolazzi P. Human polyomaviruses identification by logic mining techniques. Virology journal, 9(1):1-6, 2012.

6. Kaji M, Watanabe A, and Aizawa H. Differences in clinical features between influenza A H1N1, A H3N2, and B in adult patients. Respirology, 8(2):231-233, 2003.

## O6 A Systems Biology Compendium for Leishmania donovani

### Bart Cuypers^1,2,3^, Pieter Meysman^1,2^, Manu Vanaerschot^3,5^, Maya Berg^3^, Hideo Imamura^3^, Jean-Claude Dujardin^3,4^, Kris Laukens^1,2^

#### ^1^Advanced Database Research and Modeling (ADReM), University of Antwerp, Antwerp, Belgium; ^2^Biomedical informatics research center Antwerp (biomina), Antwerp, Belgium; ^3^Molecular Parasitology Unit, Department of Biomedical Sciences, Institute of Tropical Medicine, Antwerp, Belgium; ^4^Department of Biomedical Sciences, University of Antwerp, Antwerp, Belgium; ^5^Department of Microbiology and Immunology, Columbia University Medical Center, New York, NY, USA

##### **Correspondence:** Bart Cuypers (bart.cuypers@uantwerpen.be) – Molecular Parasitology Unit, Department of Biomedical Sciences, Institute of Tropical Medicine, Antwerp, Belgium

Jean-Claude Dujardin and Kris Laukens contributed equally to this work.

**Background**

The protozoan parasite *Leishmania donovani* is the cause of visceral leishmaniasis in the Indian subcontinent and poses a threat to public health due to increasing drug resistance. Only little is known about its peculiar molecular biology and the ‘omics integration efforts conducted so far are very limited. Here we present an integratory database that contains all genomics, transcriptomics, proteomics and metabolomics experiments that are currently publicly available for *Leishmania donovani*. In addition, the user interface contains new analysis tools that uses powerful pattern mining strategies like frequent itemset mining to allow the linking of results from different ‘omics layers in new datasets.

**Methods**

We developed a user friendly tool to crosslink all existing *L. donovani* –omics experiments. Genomics, transcriptomics, proteomics, metabolomics and phenotypic data were collected and added to a MySQL database compendium, further complemented with publicly available data. Relations between different ‘omics layers were explicitly defined and provided with a level of confidence. Python scripts were developed to preprocess, analyse and import the data. To allow comparability between different experiments the principles of the COLOMBOS bacterial expression compendium were adapted [1].

Next to this vast data resource, a set of integrative data-analysis tools was developed based on data mining strategies. For example: One tool uses frequent itemset mining algorithms to detect which proteins and metabolites frequently exhibit the same behaviour under different conditions. Another tool converts several –omics layers to a network format that can be opened in Cytoscape [2] thus be the basis for network analysis. Django and Twitter Bootstrap frameworks were used to create a web portal to make the tools accessible to any *Leishmania* researcher.

**Results**

Excellent public gene, protein and metabolite annotation databases are already available for *Leishmania* (e.g. TriTrypDB [3] and GeneDB [4]). However, the added value of our tool is that it links these annotation data to ‘omics experiments that are either provided by the user, or publicly available. New experiments can quickly be preprocessed, analysed and integrated in the database via its Python back end. Using the compendium and its tools, we characterized the development and drug-resistance of *Leishmania donovani* in a system biology context. The genomes of more than 200 strains were examined for associations with phenotypical features and a subset was linked to transcriptomics, proteomics and metabolomics results. The compendium and its scripts were designed to be generic and can therefore be used for other organisms with only minor adaptions to the original setup.

**References**

1. Meysman P, Sonego P, Bianco L, Fu Q, Ledezma-Tejeida D, Gama-Castro S, Liebens V, Michiels J, Laukens K, Marchal K et al: COLOMBOS v2.0: an ever expanding collection of bacterial expression compendia. Nucleic acids research 2014, 42(Database issue):D649-653.

2. Shannon P, Markiel A, Ozier O, Baliga NS, Wang JT, Ramage D, Amin N, Schwikowski B, Ideker T: Cytoscape: a software environment for integrated models of biomolecular interaction networks. Genome research 2003, 13(11):2498-2504.

3. Aslett M, Aurrecoechea C, Berriman M, Brestelli J, Brunk BP, Carrington M, Depledge DP, Fischer S, Gajria B, Gao X et al: TriTrypDB: a functional genomic resource for the Trypanosomatidae. Nucleic acids research 2010, 38(Database issue):D457-462.

4. Logan-Klumpler FJ, De Silva N, Boehme U, Rogers MB, Velarde G, McQuillan JA, Carver T, Aslett M, Olsen C, Subramanian S et al: GeneDB--an annotation database for pathogens. Nucleic acids research 2012, 40(Database issue):D98-108.

## O7 Unravelling signal coordination from large scale phosphorylation kinetic data

### Westa Domanova^1,2^, James R. Krycer^1,3^, Rima Chaudhuri^1,3^, Pengyi Yang^6^, Fatemeh Vafaee^1,5^, Daniel J. Fazakerley^1,3^, Sean J. Humphrey^7^, David E. James^1,3,4^, Zdenka Kuncic^1,2^

#### ^1^Charles Perkins Centre, The University of Sydney, NSW 2006, Australia; ^2^School of Physics, The University of Sydney, Sydney, NSW 2006, Australia; ^3^School of Molecular Bioscience, The University of Sydney, NSW 2006, Australia; ^4^Sydney Medical School, The University of Sydney, NSW 2006, Australia; ^5^School of Mathematics and Statistics, The University of Sydney, NSW 2006, Australia; ^6^NIH/NIEHS, NC 27709, USA; ^7^Department of Proteomics and Signal Transduction, Max Planck Institute for Biochemistry, Martinsried, 82152, Germany

##### **Correspondence:** David E. James – Sydney Medical School, The University of Sydney, NSW 2006, Australia; Zdenka Kuncic (zdenka.kuncic@sydney.edu.au) – School of Physics, The University of Sydney, Sydney, NSW 2006, Australia

Westa Domanova and James R. Krycer contributed equally to this work.

**Background**

A growing body of evidence is emerging that the temporal behavior of cellular signaling molecules controls biological responses. Phosphorylation, one of the most prevalent signaling modifications, occurs rapidly in response to environmental changes. Our understanding of the signaling network is incomplete because the kinase for the majority of phosphorylation substrates remains unknown. To elucidate the underlying topology of signaling cascades from high-throughput data, we need to be able to predict kinase substrate relationships. Currently, prediction algorithms ignore the crucial biological context of kinase substrate relationships.

**Methods**

To address this we consider temporal behaviours: given that phosphorylation events occur in a coordinated way, with some kinases being active before others, we predict site-specific kinase substrate relationships from large scale in vivo experiments. First, we used available phosphorylation databases to construct a publicly-accessible repository of experimentally-predicted kinase substrate relationships. Next, based on the substrates reported for each kinase in this database, we identify how the kinase activities change over time in temporal datasets.

**Results**

Applying this to an insulin-stimulated phosphorylation screen we were able to distinguish between the substrates of AKT and RPS6KB1, two kinases with the same consensus motif, and identified IRS-1-S270 as a novel putative AKT site. We subsequently used our ssKSR-LIVE algorithm to predict novel substrates for the kinases driving insulin signaling, shedding light on their role in driving insulin-stimulated biological processes. This algorithm can be applied to other high-throughput screens of signal transduction, and thus can be used to improve our understanding of complex diseases caused by dysregulated signalling, including cancer and type 2 diabetes.

